# Trial on Refinement of Early stage non-small cell lung cancer. Adjuvant chemotherapy with pemetrexed and cisplatin versus vinorelbine and cisplatin: The TREAT protocol

**DOI:** 10.1186/1471-2407-7-77

**Published:** 2007-05-08

**Authors:** Michael Kreuter, Johan Vansteenkiste, Frank Griesinger, Hans Hoffmann, Hendrik Dienemann, Paul De Leyn, Michael Thomas

**Affiliations:** 1 Department of Medicine/Thoracic Oncology, Thoraxklinik at the University of Heidelberg, Amalienstr. 5, 69126 Heidelberg, Germany; 2 Department of Pulmonology, University Hospital and Leuven Lung Cancer Group, Herestraat 49, 3000 Leuven, Belgium; 3 Department of Oncology, Piushospital Oldenburg, Georgstraße 12, 26121 Oldenburg, Germany; 4 Department of Thoracic Surgery, Thoraxklinik at the University of Heidelberg, Amalienstr. 5, 69126 Heidelberg, Germany; 5 Department of Thoracic Surgery, University Hospital and Leuven Lung Cancer Group, Herestraat 49, 3000 Leuven, Belgium

## Abstract

**Background:**

Adjuvant chemotherapy has been proven to be beneficial for patients with early stage non-small cell lung cancer. However, toxicity and insufficient dose delivery have been critical issues with the chemotherapy used. Doublet regimens with pemetrexed, a multi-target folate inhibitor, and platin show clear activity in non-small cell lung cancer and are well tolerated with low toxicity rates and excellent delivery.

**Methods/Design:**

In this prospective, multi-center, open label randomized phase II study, patients with pathologically confirmed non-small cell lung cancer, stage IB, IIA, IIB, T3N1 will be randomized after complete tumor resection either to 4 cycles of the standard adjuvant vinorelbine and cisplatin regimen from the published phase III data, or to 4 cycles of pemetrexed 500 mg/m2 d1 and cisplatin 75 mg/m2 d1, q 3 weeks. Primary objective is to compare the clinical feasibility of these cisplatin doublets defined as non-occurrence of grade 4 neutropenia and/or thrombocytopenia > 7 days or bleeding, grade 3/4 febrile neutropenia and/or infection, grade 3/4 non-hematological toxicity, non-acceptance leading to premature withdrawal and no cancer or therapy related death. Secondary parameters are efficacy (time to relapse, overall survival) and drug delivery. Parameters of safety are hematologic and non-hematologic toxicity of both arms.

**Discussion:**

The TREAT trial was designed to evaluate the clinical feasibility, i.e. rate of patients without dose limiting toxicities or premature treatment withdrawal or death of the combination of cisplatin and pemetrexed as well as the published phase III regimen of cisplatin and vinorelbine. Hypothesis of the study is that reduced toxicities might improve the feasibility of drug delivery, compliance and the convenience of treatment for the patient and perhaps survival.

**Trial Registration:**

Clinicaltrials.gov NCT00349089

## Background

Non-small cell lung cancer (NSCLC) accounts for the largest number of cancer deaths annually, worldwide [1]. Of these, about 30 % are early stage patients (stage I and II). For this group of patients, radical surgery with mediastinal lymph node dissection has been the mainstay of therapy with a reasonable curative option. However, 5-year survival rates for patients with pathologically staged IA-IIB disease are ranging from 67% to 39% [2]. Following surgery, distant recurrence is the most common form of relapse and eventual cause of death. Assuming that these recurrences are due to occult micrometastases at the time of surgery, trials on adjuvant systemic therapy have been performed in an attempt to reduce the risk of recurrence and to improve survival. Historically, early randomized trials of postoperative chemotherapy failed to demonstrate a consistent benefit. A meta-analysis of randomized trials compared surgery alone with additional chemotherapy in stage I-IIIA NSCLC suggesting an absolute survival benefit of 5% at 5 years for cisplatin-containing chemotherapy regimens. However, statistical significance was not reached (overall hazard-ratio (HR) of 0.87, p = 0.08) [3]. This finding prompted renewed interest in studying the beneficial effect of adjuvant chemotherapy in NSCLC.

In some of the recently published trials a clear benefit of adjuvant chemotherapy in early stage NSCLC could not be achieved [4-6]. In marked contrast to these studies, three recent, big randomized trials on early stage NSCLC patients with modern platin-based two-drug chemotherapy-regimens revealed a significant advantage for overall or relapse free survival for chemotherapeutically treated patients [7-9]. The majority of patients in the adjuvant treatment setting received a combination of cisplatin and vinorelbine. A pooled analysis of five big randomized studies demonstrated that adjuvant cisplatin-based chemotherapy improves survival in patients with NSCLC significantly (overall HR of death 0,89, p < 0,005) corresponding to a survival benefit of 4.2% for all stages [10]. However, toxicity and inadequate dose delivery have been critical issues in all trials performed so far. Grade 3/4 toxicities are observed up to 73% [11] with rates of neutropenic fever up to 7% [8]. Up to 77% of the patients had at least one dose reduction or omission and 55% required one dose delay or more, most related to neutropenia. Only about 50% of patients randomized on the combination of cisplatin and vinorelbine received the intended dose of vinorelbine and only 50% of patients completed all four cycles of chemotherapy [8,9,12]. Reasons for discontinuation of chemotherapy were mainly patient refusal combined with toxicity while disease progression or intercurrent illness were uncommon reasons [12].

Pemetrexed, a multi-target folate antimetabolite, shows clear activity in non-small cell lung cancer. In a phase III study [13] for patients with previously treated advanced non-small cell lung cancer, the efficacy of single-agent pemetrexed, as determined by overall survival, was similar to that of docetaxel. Results from Phase II studies have shown that the efficacy of a platinum based combination therapy with pemetrexed is similar to other standard platinum doublets, with response rates of 27% to 45% and median survivals of 8.9 to 10.9 months [14-18]. This doublet can be delivered easily and is well tolerated. Furthermore, it results in a 25% rate of grade 3/4 neutropenia only and the incidence of febrile neutropenia was < 1% in vitamin supplemented patients. Dose reductions occur only in 2–4% of the patients and dose delivery is excellent with delivery rates of pemetrexed up to 95% [13,14,19].

Therefore, it seems reasonable to test a less toxic regimen also in early stages after complete (R0) resection of the tumor, where reduced toxicities might improve the feasibility of drug delivery, compliance and the convenience of treatment for the patient and hence perhaps survival.

The main purpose of this randomized phase II trial, the TREAT protocol, is to evaluate the clinical feasibility – in terms of patients without dose limiting toxicities or premature treatment withdrawal or death – of the combination of cisplatin and pemetrexed as well as the combination of cisplatin and vinorelbine.

## Methods/Design

### Trial organization

TREAT has been designed by the AIO Lung Cancer Study Group, Germany and the LLCG Leuven Lung Cancer Group, Belgium under responsibility of the Clinic for thoracic diseases at the University of Heidelberg, Germany. The trial is an investigator initiated trial.

### Coordination

The trial is coordinated by the Department of Medicine/Thoracic Oncology, Clinic for thoracic diseases at the University of Heidelberg, Germany in cooperation with the Respiratory Oncology Unit (Pulmonology) and Leuven Lung Cancer Group, Catholic University, Leuven, Belgium and the Department of Hematology and Oncology, University of Goettingen, Germany. The Department of Medicine/Thoracic Oncology, Clinic for thoracic diseases at the University of Heidelberg is responsible for overall trial management, trial registration (ClinicalTrials.gov Identifier: NCT00349089, EudraCT Number 2005-004840-30), database management, quality assurance including monitoring, reporting and for the scientific program of all trial related meetings supported by Ingenix Pharmaceutical Services (Deutschland) GmbH/i3 research | SKM Oncology, Germany.

### Investigators

Patients will be recruited in the following centers: *Germany*: Departments of Medicine/Thoracic Oncology and of Thoracic Surgery, Thoraxklinik at the University of Heidelberg; Departments of Hematology and Oncology and of Thoracic and Cardiovascular Surgery, University of Goettingen; Department of Medicine/Thoracic Oncology Klinik Löwenstein, Löwenstein; Departments of Medicine/Thoracic Oncology and of Thoracic Surgery, Helios-Klinikum Emil von Behring, Berlin; Departments of Medicine/Pneumology and of Thoracic Surgery, Klinikum Bremen-Ost, Bremen; Westdeutsches Tumorzentrum, Essen; Departments of Medicine/Hematology and Oncology and of Thoracic Surgery, Dr. Horst Schmidt Klinik, Wiesbaden; Department of Medicine/Pneumology, Klinikum der Universität München; Departments of Medicine/Thoracic Oncology and of Thoracic Surgery, Lungenzentrum Großhansdorf, Großhansdorf; Departments of Medicine/Pneumology and of Thoracic Surgery, Lungenklinik Hemer, Hemer; *Belgium*: Department of Pulmonology (Respiratory Oncology Unit), University Hospital Gasthuisberg, Leuven, Department of Pneumology, Ziekenhuis Oost Limburg, Genk; Department of Pneumology, CHU Sart Tilman, Liege, Department of Thoracic Surgery, University Hospital and Leuven Lung Cancer Group, Leuven, *Luxembourg*: Departments of Hematology-Oncology and of Thoracic Surgery, Centre Hospitalier Luxembourg, Luxembourg. All investigators are experienced oncologists in the field of medical und thoracic oncology.

### Adverse events committee

This committee consists of 2 independent physicians (medical oncologist) and decides on the final diagnostic classification of critical clinical events. For all serious adverse events the documentation and relevant patient data are verified by the coordinating personnel before submitting the data to the Adverse Events Committee for diagnostic classification.

Analysis of safety related data is performed with respect to frequency of:

• Serious Adverse Events and Adverse Events stratified by organ-system

• Adverse Events stratified by severity

• Adverse Events stratified by causality.

Patient toxicities will be assessed using the NCI Common Toxicity Criteria [27].

A serious adverse event is defined as an AE occurring during any study phase and at any dose of the investigational product that fulfils one or more of the following criteria:

• results in death

• is immediately life-threatening

• requires in-subject hospitalization or prolongation of existing hospitalization

• results in persistent or severe disability, incapacity or cancer (other than the cancer diagnosed prior to enrolment in the study)

• is a congenital abnormality or birth defect

• is an important medical event that may jeopardize the subject or may require medical intervention to prevent one of the outcomes listed above

• or is significant for any other reason

Any serious adverse event experienced by the patient which might be related to the study drugs will be reported by the CRO to the sponsor, to Eli Lilly and Company for pemetrexed and to Medac GmbH for vinorelbine and for cisplatin. In addition, adverse events will be reported to regulatory authorities according to the definitions and timelines specified in the local laws and regulations. The Serious Adverse Event must be followed up until resolution, follow-up documentation has to be provided by the center.

### On-site monitoring

During recruitment of patients monitoring on site is performed according to good clinical practice (GCP) guidelines. The data management will be performed by Ingenix Pharmaceutical Services (Deutschland) GmbH/i3 research | SKM Oncology, Germany.

### Ethics, informed consent and safety

The final protocol was approved by the ethics committee of the University of Heidelberg, Germany [B28] and the Bundesinstitut für Arzneimittel und Medizinprodukte (BfArM-registration number 4031656). This study complies with the Helsinki Declaration in its recent German version, the Medical Association's professional code of conduct, the principles of Good Clinical Practice (GCP) guidelines and the Federal Data Protection Act. The trial will also be carried out in keeping with local legal and regulatory requirements. The medical secrecy and the Federal Data Protection Act will be followed.

Written informed consent is obtained from each patient in oral and written form before inclusion in the trial and the nature, scope, and possible consequences of the trial have been explained by a physician. The investigator will not undertake any measures specifically required only for the clinical trial until valid consent has been obtained.

### Study design

The TREAT study is a prospective, multi-center, open label, randomized phase II study determining the clinical feasibility in terms of toxicity of 4 cycles of adjuvant chemotherapy with pemetrexed and cisplatin versus vinorelbine and cisplatin in patients with non-small lung cancer stage IB, IIA, IIB and T3N1 (without need for further radiotherapy).

Randomization will be balanced between treatment arms according to the following factors:

• Center

• Nodal Status (N0 vs. N1)

• Surgical procedure (lobectomy vs. pneumonectomy)

The primary objective is to determine the **clinical feasibility rate (CFR) **of 4 cycles of adjuvant chemotherapy with Pemetrexed and Cisplatin vs. Vinorelbine and Cisplatin in patients with NSCLC stage IB, IIA, IIB and T3N1 (without need for further radiotherapy). Treatment is considered to have clinical feasibility if dose limiting toxicity (DLT) will not be observed, and no non-acceptance by the patient leading to premature withdrawal, and no death due to cancer or cancer therapy will occur. DLTs are defined as:

• Grade 4 neutropenia more than 7 days

• Grade 4 thrombocytopenia more than 7 days

• Grade 3/4 neutropenia with fever (i.e. > 38.5°C on at least 2 occasions in 24 hours time) and/or infection (i.e. documented by either culture or imaging method)

• Any grade thrombocytopenia with bleeding

• Grade 3/4 non-hematological toxicity possibly or probably related to the chemotherapy (except for nausea/vomiting/hair loss)

**Secondary objectives **are to determine and compare the drug delivery between both treatment arms, the time to treatment failure, the relapse free survival, the overall survival, the distant metastases free survival, local relapse free survival, the localization of relapse, the dose delivery, and safety.

### Statistical design

This phase II trial design is based on the following assumptions: the experimental therapy arm would be rated as unacceptable, if the actual feasibility rate (= 1 – withdrawal/DLT rate) was 65 % or lower. On the other hand, the therapy would be considered to be a promising candidate for further development, if the true feasibility rate amounted to 80% or more. Probability to accept the experimental therapy as well tolerable, in spite of a *true *feasibility rate of < 65% (i.e. withdrawal/DLT rate > 35%): 5% (type I error). Probability to reject the experimental therapy as not sufficiently feasible (< 65%), although the *true *feasibility rate is promising (> 80%): 20% (type II error, corresponding to a power of 80%). According to these parameters, and using the variant out of the class of optimal two-stage designs by Simon [20] that leads to the lowest maximum number of patients required (minimax approach), **n = 18 patients **evaluable for feasibility have to be recruited in the first stage. The combination will be rejected, if three or more of these patients fulfil the criterion of non-feasibility. In the second step, further patients will be recruited up to a total number of **67 cases**. The final conclusion of the trial will depend on the definite feasibility rate (and its confidence interval), the respective findings in the vinorelbine/cisplatin reference arm, the achieved level of drug delivery as well as the complete information on type, frequency and severity of toxicities. As a similar number of patients is to be recruited to the standard arm, a total number of **134 patients **is required. All parameters will be evaluated in an explorative or descriptive manner, providing means, medians, ranges, standard deviations and/or confidence intervals. If p values are calculated (e.g. in subgroup comparisons or across treatment arms), they will be presented explicitly without referring to hypotheses or a significance level. Usually, no error adjustment for multiple testing will be performed. Thus the p values will reflect the comparison-wise error and not the experiment-wise error. All p values will be two-sided if not otherwise stated. The statistical methods described in this section are suited for the data and distributions usually expected in this type of trials. The suitability will be checked after data entry. If necessary, the statistical method will be modified accordingly. Feasibility, toxicity and other event rates at pre-specified time points are calculated, providing confidence intervals. In case of comparison between patient groups, these rates will be analyzed by Fisher's exact test, χ^2 ^test or Mantel-Haenszel test (or trend test according to Cochran/Armitage), respectively. Event related data like relapse-free or overall survival will be estimated by the product limit method by KAPLAN and MEIER and compared using the logrank test. If the Peto logrank test is not appropriate because of violation of the proportional hazard assumption, Gehan's generalization of the Wilcoxon rank sum test for censored data will be applied. If appropriate, prognostic strata will be taken into account. The methods mentioned above are likewise suitable for the univariate evaluation of prognostic factors. Multivariate analyses may be performed by suitable regression models (COX proportional hazard regression model, logistic regression).

### Patient selection

In order to be included in the TREAT trial, patients are required to have histopathologically confirmed diagnosis of non-small cell lung cancer (NSCLC), pathologic stage IB, IIA, IIB or T3N1 (without need for further radiotherapy). Tumor had to be resected completely without detectable residual tumor including negative margins (R0) and systematic intraoperative dissection of mediastinal lymph nodes according to the guidelines of the British thoracic society had to be performed [21]. All accessible hilar and mediastinal lymph nodes should be removed for pathologic evaluation. Study drug administration should only be administered to patients with full recovery after surgery and is to begin on d28 to d42 postoperatively. Histological tumor types which are eligible are squamous cell carcinoma, adenocarcinoma (including adenocarcinomas with bronchioloalveolar differentiation), large cell carcinoma (excluding tumors with slight areas of small cell carcinoma) and mixed cell carcinoma without small cell fraction.

The following **inclusion criteria **are required: Provision of informed consent according to local regulatory requirements for participation in the study, Age = 18 years; < 75 Years, Karnofsky Performance Status ≥ 80% or ECOG ≤ 1, adequate hematological laboratory parameters (Hemoglobin ≥ 10 g/dl, ANC ≥ 1500/μl, Platelets ≥ 100000/μl), adequate hepatic laboratory parameters (Bilirubin ≤ 1.5 × UNL, ASAT/ALAT ≤ 2 × UNL), adequate renal laboratory parameters (Creatinine ≤ 1,5 mg/dl and calculated Creatinine Clearance ≥ 60 ml/min). Furthermore cardiac function allowing cisplatin chemotherapy (in case of doubt echocardiography is mandatory documenting LVEF > 49%), electrocardiogram without significant cardiac arrhythmia, FEV1 ≥ 1.2 l post-operatively, respiratory function not impeding cisplatin-based chemotherapy assessed by either absolute DLCO or capillary/arterial BGA in resting condition (absolute DLCO > 40 % or pO_2 _> 60 mmHg in resting condition), agreement by the patient to use an effective method of contraception, negative pregnancy test for women of childbearing potential unless they are postmenopausal at baseline (postmenopausal women must have been amenorrheic at least for 12 months to be considered of non childbearing potential).

Patients are **not eligible **with the presence of a Pancoast tumor, involvement of N2/N3 lymph nodes and/or distant metastases. The following histological tumor types are excluded: pure bronchioloalveolar carcinoma, mixed cell carcinoma with small cell fractions, large cell carcinoma with areas of small cell carcinoma. Further exclusion criteria are pregnancy or lactation period, other co-existing malignancies or malignancies diagnosed within the last 5 years with the exception of a CIS of the cervix or non-melanomatous skin cancer, radio- and/or chemotherapy within the last five years, concurrent administration of any other antitumor therapy, non-compliance with vitamin (folic acid and vitamin B12) intake or to whom administration is not possible, hypersensitivity to pemetrexed or to any of the excipients of Alimta™, to cisplatin or to any other platinum compound and/or to vinorelbin or to any other vinca-alkaloid. Patients who have previously completed or been withdrawn from this study or any other study with the respective medication in this study, have had treatment with an investigational new drug, currently or within the last 30 days, and/or participation in another clinical trial, currently or during the last 12 weeks, and/or previous participation in this study, history of a psychological illness or condition such as to interfere with the patient's ability to understand the requirements of the study, with any clinically significant disease that in the opinion of the investigator is likely to put the patient at risk or to interfere with the evaluation of the patient's safety and of the study outcome (this includes, but is not limited to: clinically significant cardiac disease [e.g. congestive heart failure, symptomatic coronary artery disease and cardiac arrhythmia not well controlled with medication] or myocardial infarction within the last 6 months, uncontrolled hypertension, interstitial pneumonia or extensive or symptomatic interstitial fibrosis of the lung, pleural effusion or ascites, which cause respiratory compromise), a history or presence of any CNS disorder or psychiatric disability judged by the investigator to be clinically significant and/or interfering with compliance, a serious concomitant systemic disorder (e.g. active infection including HIV) that in the opinion of the investigator would compromise the patient's ability to complete the study, post-operative complications or other surgery-related conditions that could interfere with a study participation, hearing function/tinnitus impeding chemotherapy with cisplatin and/or vinorelbine, alcohol and/or drug abuse, inability to interrupt high dose salicylates (like aspirin) or other non-steroidal anti-inflammatory drugs (NSAID's) for a 5-day period starting 2 days before administration of Pemetrexed (8-day period for long-acting agents such as piroxicam) and patients with organ allografts, neurologic disorders and who cannot be regularly observed for psychological, sociological or geographical reasons or other concomitant conditions not permitting adequate follow-up and compliance to the protocol are not eligible. Patients with sero(pneumo)-thorax after pneumonectomy or lobectomy will not be excluded. Those patients must be monitored for toxicity closely for any other active or uncontrolled infection.

### Work-up

The following assessments will be performed before, during and after the study (Table 2). Optimal preoperative staging should have consisted of the following procedures and should have been assured at least in a time interval starting **28 **days prior to surgery: CT scan of the thorax and abdomen including adrenal glands and the right lower liver margin (in case adrenal glands are not available further CT scan of the abdomen is mandatory; if only right lower liver margin is not available assessment by either CT scan of abdomen or abdominal ultrasound should be performed), MRT (preferably) or CT scan of the skull and bone scintigraphy (if a FDG-PET scan has been performed bone scintigraphy can be omitted). Not more than **14 **days prior to the start of chemotherapy, for all patients the following baseline parameters will be assessed: patient demography, clinical examination including physical examination, height, weight, vital signs, Karnofsky or ECOG Performance Status, assessment of the neurological status, existing signs and symptoms, medical history (including concurrent illnesses) and specific details on the diagnosis of non-small cell lung cancer (NSCLC), previous anti-cancer therapy and their outcome, concomitant medication, laboratory assessment including hematological parameters (hemoglobin, WBC, ANC, platelets), electrolytes (Na, K, Ca), hepatic parameters (total bilirubin, ASAT, ALAT, AP, gGT, LDH), renal parameters (creatinine, calculated creatinine clearance, Urea, uric acid), coagulation parameters (Quick, PTT, Fibrinogen), and pregnancy test for women with childbearing potential. Furthermore chest-X-ray, electrocardiography and optional echocardiograph (mandatory only in case of doubt whether cardiac function allows cisplatin chemotherapy) will be performed. Pulmonary function will be assessed by FEV1, vital capacity and total capacity and by either absolute DLCO or capillary/arterial BGA in resting condition with absolute DLCO > 40 % or pO_2 _> 60 mmHg in resting condition. Patients with clinical suspicion of altered hearing capability or symptoms, e.g. tinnitus, should undergo further evaluation by audiometry. If preoperative staging had not comprised all mandatory procedures it should be completed as outlined above before registration.

Every patient must provide a written informed consent to the trial procedures. The patient must be informed verbally and by the provided patient information by the investigator, or a person designated by the investigator if permitted by local regulations, before informed consent is obtained.

### Study phase

In an initial study phase of 4 months of patient enrollment, 36 patients (i.e. 18 in each treatment arm) will be accrued to confirm feasibility. In the second step, further patients will be recruited up to a total number of 134 (i.e. 67 cases per treatment arm).

### Period of Adjuvant Chemotherapy

A cycle is a treatment period of 21 days for Pemetrexed and Cisplatin (Arm A) or 28 days for Vinorelbine and Cisplatin (Arm B). Study drug administration is to begin on d28 to d42 after R0 resection of the tumor and within 14 days after randomization.

#### Arm A : Pemetrexed and Cisplatin combination

For patients in arm pemetrexed/cisplatin, folic acid (350–1000 μg) must be given daily beginning approximately 5–7 days prior to first dose of pemetrexed and continuing daily until 3 weeks after the last dose of study therapy. Vitamin B12 (1000 μg) will be administered as an intramuscular injection approximately 1 to 2 weeks prior to first dose of pemetrexed and repeated approximately every 9 weeks until 3 weeks after the last dose of study therapy. Dexamethasone (4 mg of oral or equivalent) given twice daily should be taken on the day before, the day of, and the day after each dose of pemetrexed, for rash prophylaxis unless medically contraindicated. Patients must receive pemetrexed at day 1 at the dose of 500 mg/m2 as an IV infusion over approximately 10 minutes. Beginning 30 minutes after the end of the pemetrexed infusion, cisplatin at a dose of 75 mg/m2 should be administered IV over approximately 60 minutes. Each institution should provide adequate hydration with cisplatin treatment, according to local habits and the approved package insert of cisplatin, and similarly in both treatment arms. A cycle is a treatment period is 21 days; a total of four cycles is intended.

#### Arm B: Vinorelbine and Cisplatin combination

Patients in arm vinorelbine and cisplatin follow the regimen as in the published phase III adjuvant study [8]: IV vinorelbine at the dose of 25 mg/m2 in combination with cisplatin at a dose of 50 mg/m2 at day 1, followed by the same schedule with IV vinorelbine at the dose of 25 mg/m2 in combination with cisplatin at a dose of 50 mg/m2 at day 8. Vinorelbine will be repeated on days 15 and 22 at the dose of 25 mg/m2. The scheduled infusion time is 6–10 minutes for vinorelbine and approximately 60 minutes for cisplatin. Each institution should provide adequate hydration with cisplatin treatment, according to local habits and the approved package insert of cisplatin, and similarly in both treatment arms. A cycle is a treatment period is 28 days; a total of four cycles is intended.

During the chemotherapy the following assessments will be performed (Table 2): clinical examination including physical examination, neurologic assessment, weight, vital signs, Karnofsky or ECOG Performance Status at the beginning of each cycle, hematological laboratory assessment (hemoglobin, WBC, ANC, platelets) at least once weekly. At the start of each cycle (3 days prior to or at least at day 1), laboratory assessment including hematological parameters (hemoglobin, WBC, ANC, platelets), electrolytes (Na, K, Ca), hepatic parameters (total bilirubin, ASAT, ALAT, AP, LDH), renal parameters (Urea, uric acid, creatinine, calculated creatinine-clearance), compliance, concomitant medication and adverse events will be recorded. In case of suspicious findings a relapse of disease has to be confirmed by further imaging.

### Toxicity management

Toxicities are classified by grade, type, duration, onset, and relationship to study treatment according to CTCAE version 3.0.

After application of chemotherapy blood count should be performed at least once weekly. In case of leukocytopenia or neutrocytopenia CTC grade 4 (leukocytes < 1.0 × 10^9^/L), antibiotic prophylaxis according to local habits is recommended. Prophylaxis should be used similarly in both treatment arms. Routine use of colony-stimulating factor (CSF) is not permitted during this study. ASCO guidelines for use of CSF should be followed [22]. Granulocyte colony stimulating factor must have been discontinued at least 24 hours prior to the start of the next chemotherapy infusion. In case of infection in neutropenia or occurrence of fever of unknown origin, further diagnostic procedures should be performed and antibiotic treatment according to the criteria of the German Paul-Ehrlich-Gesellschaft should be initiated [23]. In case of thrombocytopenia CTC grade 4 the patient will be discontinued from the study. In the event of CTC Grade 3 or 4 diarrhea, the following supportive measures are allowed: hydration, octreotide, and antidiarrheals. If diarrhea is severe (requiring intravenous rehydration) and/or associated with fever or severe neutropenia (Grade 3 or 4), broad-spectrum antibiotics must be prescribed. Patients with severe diarrhea or any diarrhea associated with severe nausea or vomiting must be hospitalized for intravenous hydration and correction of electrolyte imbalances. Patients with CTC grade 3 or 4 diarrhea will be discontinued from the study. Professional supervised oral care protocols that include patient education in an attempt to reduce the severity of mucositis from chemotherapy is highly advised to all patients. In case of mucositis related pain, symptomatic therapy according to WHO guidelines should be performed. Based on their proposed mechanisms, antimicrobial agents, such as combined polymyxin E, tobramycin, and amphotericin or single-agent iseganan, appear to have no associated mechanistic rationale for the prevention of mucositis and probably could provide benefit only for patients with late-stage ulcerative mucositis, in which bacterial superinfection occurs. The use of amphotericin suspension at clinical signs of mucosal soor and at mucositis Grad 3/4 is recommended. For patients receiving pemetrexed leucovorin should be considered. Patients with CTC grade 3 or 4 mucositis will be discontinued from the study.

### Dose adjustments, safety and discontinuation of treatment

The dose reductions are limited to a preset pattern, and eliminate the need to make any calculations or resolve conflicting recommendations if two or more toxicities occur within the same cycle. It also ensures that therapeutic chemotherapy doses will be administered in all treatment cycles.

Patients should be instructed to report any toxicity that occurs during drug administration of each treatment course and in the period between cycles.

Treatment will be modified in case of hematological and/or non-hematological toxicities. All dose adjustments will be made according to the system showing the greatest degree of toxicities. Toxicities will be graded according to the NCI Common Toxicity criteria (CTCAE version 3.0). No dose re-escalation will be performed after dose reduction. If the study treatment cannot be administered after an additional 2 weeks delay because of any toxicity, it should be definitively discontinued.

***On day 1 ***of each cycle either in arm A or in arm B, the following criteria have to be met for the administration of both cisplatin and pemetrexed or vinorelbine, respectively: ANC = 1,500/μl, Platelets = 100,000/μl, Serum creatinine < 1,5 mg/dl and calculated creatinine clearance = 60 ml/min, no other grade = 2 toxicity (except for clinically non-relevant AEs such as alopecia, altered taste, nausea, vomiting). If these criteria are not met, drug administration has to be delayed up to 1 week to allow for recovery. If a delay of more than 14 days due to toxicity is necessary, the patient is to be discontinued from the study.

***On day 8 ***for the administration of both cisplatin and vinorelbine, the following criteria have to be met : ANC = 1,500/μl, Platelets = 100,000/μl, Serum creatinine < 1.5 mg/dl and creatinine clearance = 60 ml/min, Bilirubin < 1.5 × UNL. If these criteria are not met, drug administration has to be delayed up to 1 week to allow for recovery. If a delay of more than 14 days due to toxicity is necessary, the patient is to be discontinued from the study.

***On day 15 and 22 ***of each cycle, the following criteria have to be met for the administration of vinorelbine (according to [8]): Neutrophils (ANC) = 1,500/μl, Platelets = 100,000/μl, Bilirubin < 1.5 × UNL, no other grade = 2 toxicity (except for clinically non-relevant AEs such as alopecia, vomiting, nausea, altered taste). If these criteria are not met, Vinorelbine administration is omitted at that day, and treatment with Vinorelbine will be resumed on day 22 or together with Cisplatin in the following next cycle, respectively.

Dose adjustments according to **hematological toxicity **at the start of a subsequent course of the therapy will be based on platelet and neutrophil nadir counts from the preceding cycle of therapy. No dose modifications will be made for anemia. ANC must be = 1,500/μl and platelets = 100,000/μl as outlined above prior to the start of any cycle. Treatment may be delayed to allow sufficient time for recovery. Upon recovery patients should be re-treated using the guidelines as outlined in table 1.

Any patient experiencing Grade 3/4 **non-hematological **toxicity (except for nausea/vomiting/hair loss) associated with therapy will be discontinued from the study.

In case of neurological and/or hearing CTC grade 2 toxicity, administration should be delayed and reassessed one week later. If toxicity has resolved at least to grade 1 therapy should be continued with 50% dose reduction for cisplatin for further administrations. In case of decrease of calculated creatinine clearance, despite adequate hydration, administration has to be delayed and reassessed one week later and if the value of creatinine clearance remains < 60 ml/min, patient has to be discontinued.

For patients who develop clinically significant pleural or peritoneal effusions (on the basis of symptoms or clinical examination) during therapy, consideration should be given to drain the effusion prior to therapy. Though, if, in the investigator's opinion, the effusion represents relapse and/or progression of disease, the patient should be discontinued from study therapy. However, disease relapse has to be confirmed by adequate imaging methods. Patients with sero(pneumo)-thorax after hemi-pneumonectomy or lobectomy will not be excluded. Those patients must be monitored for toxicity closely.

Dose reductions for hepatic dysfunction will be based on bilirubine and/or transaminase values. For bilirubin values 1.5–3 × UNL and ASAT/ALAT < 5 × UNL, vinorelbine has to be reduced to 50%. For Grade 3/4 hepatic toxicity the patient has to be discontinued from study.

### Premature Withdrawal

Patients have the right to withdraw from the study at any time for any reason. The investigator also has the right to withdraw patients from the study in the event of intercurrent illness, adverse events, protocol violations, administrative reasons or other reasons. Discontinuation of a patient should be based upon one of the objective toxicity criteria. The following reasons can lead to premature withdrawal from therapy of a patient: major protocol violation, Non-compliance of a patient with protocol procedures, unacceptable toxicity according to the protocol, unacceptable toxicity as perceived by the patient (refusal to continue), withdrawal of consent by the patient, lost to follow-up, pregnancy, medical decision by the investigator, appropriate evaluation demonstrates recurrence of disease. The reason for withdrawal of a patient needs to be documented and the study coordinating center has to be informed immediately. Patients who have been withdrawn from therapy will have to be further documented for follow-up.

### Surgery

Surgery will be performed prior to chemotherapy. The surgical principles follow the guidelines of oncologic surgery. The tumor and all intrapulmonary lymphatic drainage must be removed completely, employing lobectomy or pneumonectomy or complex resections, if necessary. En bloc resection of closely adjacent or invaded structures is preferential to a discontinuous resection. Resection margins should be assessed by frozen section analysis whenever possible; this includes bronchial, vascular and other margins with close proximity to the tumor if necessary to obtain radical resection. Re-excision is preferred whenever possible, if positive resection margins are encountered. To achieve uncomplicated bronchus- and trachealhealing stump or anastomoses (sleeve resection) can be covered with viable tissue (pedicled pleural, pericardial, intercostal muscle flap or others). Considering the criteria of functional operability, the aim is to obtain R0-resection. All accessible hilar and mediastinal lymph nodes should be removed for pathologic evaluation, using the technique of mediastinal lymph node dissection. Technique of mediastinal lymph node dissection is recommended according to the guidelines of the British Thoracic Society [21,24]. Lymph nodes are to be identified and properly labelled by the surgeon. Tumor infiltration of lymph nodes may not be apparent and is recommended to be diligently sought. Microscopic assessment is required to accurately determine the N-status and should be performed as described in [25]. Complete mediastinal lymph node dissection is defined as when all tissue containing lymph nodes is removed at all levels accessible within the operation. Reflecting lymph node involvement, R0-resection is defined by the absence of tumor infiltration in the most distal lymph node level removed according to [26]. If possible, the most distant level should be the upper-paratracheal lymph nodes (Level 2). The natural course of a developing sero(pneumo)-thorax in case of pneumonectomy or lobectomy is not regarded as a exclusion criteria. However, patients must be followed up closely for developing toxicities.

### Radiotherapy

Radiotherapy is not planned.

### Maintenance Therapy and Follow Up Period

No maintenance therapy is allowed. Follow-up visits are planned starting at 30 days after the end of the last chemotherapy cycle and afterwards in 3 monthly intervals for the first 2 years. In the 3^rd ^year patients will be followed up in 6 monthly intervals (Table 2).

The follow up visits comprise clinical examination including physical examination, neurologic assessment, weight, vital signs, Karnofsky or ECOG Performance Status, laboratory assessment including hematological parameters (hemoglobin, WBC, ANC, platelets), hepatic parameters (total bilirubin, ASAT, ALAT, AP, LDH), chest X-ray, with further examinations in case of clinical symptoms, to confirm relapse by imaging techniques, abdominal ultrasound (optional; left to the discretion of the participating center. Center must have determined follow up procedure with abdominal ultrasound before initiation of the study in the center), concomitant medication. Additional assessments in the 1^st ^follow up at 30 days after the end of the last chemotherapy cycle comprise assessment of FEV, of vital and total capacity, capillary or arterial BGA under resting conditions, absolute DLCO and adverse events. Further examinations in case of clinical symptoms to confirm relapse by imaging techniques

### Trial duration

Individual participation is completed either three years after enrolment or death of the patient. Duration of the study is about 4 years, 6 months

## Discussion

Definitive surgery has been the mainstay of therapy of patients with early stage non-small cell lung cancer. Outcome in this group of patients was improved by administering adjuvant chemotherapy with systemic platinum-based chemotherapy regimens [7-10]. However, the combination of cisplatin and vinorelbine resulted in rates of grade 3/4 neutropenia of around 75%, rates of febrile neutropenia of up to 12.5% and rates of treatment related death of 1–2%. Up to 77% of the patients had at least one dose reduction or omission and 55% required one dose delay or more, most related to neutropenia. Only about 50 % of patients randomized on the combination of cisplatin and vinorelbine received the intended dose of vinorelbine (dose reduction mainly due to toxicity) and only 50% of patients completed all four cycles of chemotherapy [8,9,12]. Particularly patients who underwent pneumonectomy were more likely to fail chemotherapy than patients with lesser extent of resection. Those patients completed chemotherapy only in 41% of cases (OR 0.34; 95% CI 0.17–0.68) [12]. These patients mainly discontinued therapy due to toxicity, with statistically significant more frequently grade 3/4 toxicities than in patients with lesser extent of surgery. Reasons for discontinuation of chemotherapy were mainly patient refusal combined with toxicity while disease progression or intercurrent illness were uncommon reasons [12]. One has to consider that relevant toxicities are associated with poor compliance and are still a major issue in clinical trials of adjuvant therapy. Furthermore one has to take into account, that toxicity especially in patients with lung resection for lung cancer might be even more critical than in patients under adjuvant treatment for other cancers. First of all due to the resection of the lung, where the occurrence of pneumonia is a severe complication – particularly after pneumonectomy, which was performed in 24–37% of patients in the adjuvant trials. Second, with a median age of 60–65 and most patients presenting with chronic obstructive lung disease the risk of pneumonia after resection, particularly after pneumonectomy should be reduced as far as possible. Therefore it seems reasonable to test a less toxic regimen also in early stages after R0 resection of the tumor, where reduced toxicities might improve the feasibility of drug delivery, compliance and the convenience of treatment for the patient and hence perhaps survival. Pemetrexed shows clear activity in non-small cell lung cancer [13] and has demonstrated efficacy in combination regimes similar to other standard platinum doublets [14-18]. The combination of platin and pemetrexed can be easily delivered, is well tolerated and only results in a 25% rate of grade 3/4 neutropenia and -in vitamin supplemented patients- the incidence of febrile neutropenia was < 1%. Dose reductions occur only in 2–4% of the patients and dose delivery of the intended pemetrexed and platin dose is excellent with dose deliveries of pemetrexed up to 95% [13,14,19]. Considering these issues there is a legitimate hope that side-effects can be minimized and that the therapy might at least be equi-effective in comparison to the chemotherapy regimens used so far. Thus, this treatment could become an option for patients with NSCLC in the adjuvant setting.

## Abbreviations

AE adverse event

ANC absolute neutrophil count

AUC area under the concentration time curve

BGA blood gas analysis

CT computed tomography

DLCO diffusion capacity for carbon monoxide

FDG-PET 18F-fluorodeoxyglucose-Positron emission tomography

FEV1 Forced expiratory volume in one second

i.m. intramuscular

MRT Magnetic resonance tomography

NSAID Non-steroidal anti-inflammatory drug

NSCLC Non-small cell lung cancer

TLC Total lung capacity

UNL Upper Normal Limit

VC Vital capacity

WBC white blood cell count

## Competing interests

MT and JV received fees for lectures and research funding by Eli Lilly and company.

## Authors' contributions

MK, JV, FG and MT planned, coordinate and conduct the study. Medical care is covered by all participating centers who are also responsible for patient recruitment. The scientific program was planned and is carried out by MK, JV, FG, HH, HD, PD, and MT. All authors read and approved the final manuscript.

## Pre-publication history

The pre-publication history for this paper can be accessed here:



## Figures and Tables

**Table 1:** Dose modifications according to hematological toxicity in previous cycle

Platelets (× 10^9^/L) Nadir	ANC (× 10^9^/L) Nadir	Percent or previous dose (both drugs)
≥ 50 and	≥ 0.5	100 %
≥ 50 and	< 0.5	75%
< 50 and	Any	50%
Any and	< 1.0 + fever of = 38.5°C^a^	75%
Recurrence of CTC Grade 3 or 4 after 2 dose reductions	Recurrence of CTC Grade 3 or 4 after 2 dose reductions	Discontinue patient from study

^a ^these criteria meet the CTCAE version 3.0 (NCI 2003) definition of febrile neutropenia

**Table 2:** Flow chart study procedures

			Adjuvant Chemotherapy Phase										
						
	Pre-study Phase	Pre-study Phase	Cycle 1		Cycle 2		Cycle 3		Cycle 4		1^st ^follow up (30 days after end of chemo-therapy)		Further Follow-Up
Treatment Arm			A	B	A	B	A	B	A	B	A	B	A &B

Timeframe (days)	-28 prior to Surgery	-14 prior to Chemotherapy start	1–21	1–28	22–42	29–56	43–63	57–84	64–84	85–112	114	142	until 3 years after CTX

day of visit	-28 prior to Surgery	-14 prior to Chemotherapy start	1	1, 8, 15, 22	22	29, 36, 43, 50	43	57, 64, 71, 78	64	85, 92, 99, 106	114	142	starting 3 month after 1^st ^follow up in Years 1 & 2: 3-monthly, then 6-monthly

**Informed Consent**		X											
Demography		X											
Pregnancy test		X^1^											
Medical history, Signs and symptoms		X											
Neurological Status		X	X		X		X		X				
Clinical Examination^2^		X^2^	X^2^		X^2^		X^2^		X^2^		X^2^		X^2^
Concomitant medication		X	X		X		X		X		X		X
Laboratory hematology		X	X^3,4^		X^3,4^		X^3,4^		X^3,4^		X		X
Laboratory hepatic		X	X^4^		X^4^		X^4^		X^4^		X		X
Laboratory renal		X	X^4^		X^4^		X^4^		X^4^				
Laboratory electrolytes		X	X^4^		X^4^		X^4^		X^4^				
Laboratory coagulation		X											
12-lead ECG		X											
Assessment of LVEF^9^		X											
Chest-X-ray		X									X^5^		X^5^
Abdomen ultrasound	X^6^										X^10^		X^10^
MRT or CT skull	X												
CT^6 ^lower liver marginliver	X												
Assessment of vital/total capacity		X									X		
Assessment of FEV1		X									X		
Assessment of absolute DLCO or BGA under resting conditions		X									X		
Bone Scintigraphy^7^	X												
Audiometry^8^		X											
Compliance		X	X		X		X		X				
Adverse events		X	X		X		X		X		X		
Follow-Up tumor assessment^5^											X		X

1 in women with child-bearing potential

2 Clinical examination including physical examination, neurologic assessment, height (height only to be assessed at baseline), weight, vital signs, Karnofsky or ECOG Performance Status at the beginning of each chemotherapy cycle.

3 hematology parameters, including Hb, platelets, WBC, ANC at least once weekly during chemotherapy phase

4 Three days prior to or at least at day 1 of each chemotherapy-cycle

5 Relapse of disease should be confirmed by imaging techniques

6 CT scan of the thorax and abdomen including adrenal glands and the right lower liver margin (in case adrenal glands are not available further CT scan of the abdomen is mandatory; if only right lower liver margin is not available assessment by either CT scan of abdomen or abdominal ultrasound should be performed)

7 If a FDG-PET scan has been performed, bone scintigraphy can be omitted

8 Optional; patients with clinical suspicion of altered hearing capability or symptoms should undergo further evaluation by audiometry

9 Echocardiography optional, mandatory only in case of doubt whether cardiac function allows Cisplatin chemotherapy

10 optional, left to the discretion of the center prior to study initiation of the center
